# Mechanically Aligned Second-Generation Medial Pivot Primary Total Knee Arthroplasty Does Not Reproduce Normal Knee Biomechanics: A Gait Analysis Study

**DOI:** 10.3390/jcm13185623

**Published:** 2024-09-22

**Authors:** Matteo La Verde, Claudio Belvedere, Eugenio Cammisa, Domenico Alesi, Alberto Fogacci, Maurizio Ortolani, Nicoletta Sileoni, Giada Lullini, Alberto Leardini, Stefano Zaffagnini, Giulio Maria Marcheggiani Muccioli

**Affiliations:** 1 II Orthopaedic and Traumatology Clinic, IRCCS Istituto Ortopedico Rizzoli—DIBINEM, University of Bologna, 40136 Bologna, Italy; matteo.laverde@ior.it (M.L.V.); domenico.alesi@ior.it (D.A.); alberto.fogacci@ior.it (A.F.); stefano.zaffagnini@ior.it (S.Z.); giulio.marcheggiani2@unibo.it (G.M.M.M.); 2 Movement Analysis Laboratory, IRCCS Istituto Ortopedico Rizzoli, 40136 Bologna, Italy; belvedere@ior.it (C.B.); maurizio.ortolani@ior.it (M.O.); nicoletta.sileoni@ior.it (N.S.); leardini@ior.it (A.L.); 3 Operational Unit of Orthopedics and Traumatology, Imola Hospital Santa Maria della Scaletta, 40026 Imola, Italy; e.cammisa@ausl.imola.bo.it; 4 IRCCS Istituto Delle Scienze Neurologiche di Bologna, UOC Medicina Riabilitativa e Neuroriabilitazione, 40124 Bologna, Italy

**Keywords:** TKA, knee arthroplasty, medial pivot, gait analysis

## Abstract

**Background:** This study aimed to evaluate post-operative lower limb function following second-generation mechanically aligned medial pivot (MP) TKA implantation. Standard gait analysis was performed to collect kinematic and kinetic data, which were then compared with physiological data from the literature obtained using the same evaluation methodology as the present study. The hypothesis was that this TKA would not fully restore normal knee and adjacent joint motion during walking. **Methods:** Our cohort comprised 15 patients consecutively enrolled from September 2019 to December 2022 who underwent primary TKA with the second-generation MP Evolution Knee System (MicroPort Orthopaedics Inc., Arlington, TN, USA). Pre-operatively and 6 months post-surgery, gait analysis during level walking was performed on all patients, as well as clinical evaluations using the Knee Society Score (KSS), the Knee Injury and Osteoarthritis Outcome Score (KOOS), and the Visual Analogue Scale (VAS). **Results:** The clinical scores improved significantly (*p* < 0.001) after surgery (pre-/post-operative KSS functional, KSS clinical, VAS, and KOOS: 51.7 ± 17.3/84 ± 18.4, 45.3 ± 16.2/74.1 ± 12.6, 6.9 ± 1.8/2.0 ± 1.9, and 33.9 ± 11.8/69.1 ± 16.5, respectively). The statistical parametric mapping (SPM) analysis between the post-operative and reference control data revealed significant differences in the initial and final 20% of the gait cycle for the rotation of the knee in the frontal and transverse planes and for the rotation of the ankle in the sagittal plane. **Conclusions:** This study shows that new-generation MP TKA with mechanical alignment does not fully restore normal gait biomechanics, particularly in knee rotational movements, indicating a need for improved surgical techniques and prosthetic designs.

## 1. Introduction

In recent years, developments in the field of knee prosthetics have provided innovative designs that aim to improve the performance and satisfaction rate of patients undergoing primary total knee arthroplasty (TKA). Among these developments, medial pivot (MP) TKA has emerged as a means to reproduce more natural knee kinematics, where the medial femoral condyle is congruent on the concave medial tibial plateau, acting as a ball-in-socket mechanism [1,2]. The tibial surface is highly congruent and asymmetric, allowing for natural rolling and sliding.

Several studies have tried to identify whether first-generation MP TKA can really reproduce the same kinematic characteristics as the native knee and achieve good clinical outcomes [3,4,5]. Beach et al. evaluated the external rotation of the MP design using motion analysis, focusing on the peculiar “screw home” movement or the relative tibiofemoral axial rotation during the last 20–30° of knee extension [4]. They found only significant differences between the MP and CR implant designs for the step ascent task; in addition, none of the implant designs exhibited a physiological “screw home” mechanism. A study of the Australian Orthopaedic Association National Joint Replacement Registry and Norwegian Arthroplasty Register from 2005 to the end of 2017 showed that first-generation MP prostheses had a higher revision risk than second-generation prostheses; this was mainly due to lysis or loosening, instability, and malalignment [6]. Miura et al., studying second-generation MP TKA, attempted to determine the kinematics and axial center of rotation during walking [7]. They found that most of the patients who underwent MP TKA showed a lateral pivot pattern during walking, highlighting how the impact of MP geometry on kinematics during walking is limited. A recent systematic review examined gait analysis studies after primary MP and posterior-stabilized (PS) TKAs, confirming that both designs were actually unable to reproduce the screw home mechanism [8]. The first-generation model of MP TKA provided a more traditional approach to knee replacement, i.e., with symmetrical tibial bases and limited sizing options, which can sometimes lead to compromises in fit and alignment. In contrast, the second-generation MP TKA now features an asymmetrical tibial base paired with a tibial insert that includes anterior and posterior medial lips. These design features are meant to limit both the anterior and posterior translation of the tibial component with respect to the femoral component. This, combined with the modified radius of curvature of the artificial femoral condyles, effectively reduces the risk of femoral slippage, which can lead to a feeling of instability in patients. Additionally, the second-generation MP TKA offers a wider range of sizes, with the option for size interchangeability between femoral and tibial components, enabling the customization of the implant to the patient’s unique anatomy. Furthermore, the second-generation version incorporates improved design elements and materials aimed at reducing wear and tear, potentially increasing the longevity of the system [9,10,11].

To date, the kinematics of the second-generation MP TKA have not been fully investigated; in fact, in the few relevant studies in the literature, functional evaluations of normal daily activities are scarce. Furthermore, no comparative studies with the normal population using instrumented gait analysis and advanced and accurate computerized techniques have been reported.

The aim of this present study was to assess the post-operative functional behavior of the lower limb after the implantation of a second-generation mechanically aligned MP TKA. To achieve this, standard gait analysis during walking was performed to collect kinematic and kinetic data and compare these data with physiological data from the literature obtained using the same evaluation methodology as the present study. The hypothesis was that a mechanically aligned second-generation MP TKA would not allow for the full restoration of the physiological motion of the knee and the neighboring joints during walking.

## 2. Material and Methods

### 2.1. Participants and Inclusion and Exclusion Criteria

Our cohort comprised 15 patients (data for which are provided in Table 1) undergoing primary TKA with a second-generation MP design. The patients were consecutively enrolled from September 2019 to December 2022 after signing an informed consent form. The patients were evaluated pre-operatively and post-operatively at the 6-month follow-up stage.

Our assessment involved the adoption of standard clinical scoring systems, as well as radiological examinations and instrumental gait analysis. The study protocol was approved by the local Institute Ethics Committee (PROT-0009896, 507/2019/Sper/IOR). The study was conducted in accordance with the Declaration of Helsinki. The participants provided written informed consent prior to participation.

The inclusion criteria were as follows: (a) aged between 50 and 75 years old, (b) primary or post-traumatic OA, (c) femoral or tibial osteonecrosis, (d) valid collateral ligaments at the affected knee, (e) varus or valgus deformity between 0 and 10°, (f) capable of giving informed consent, and (g) compliant with rehabilitation. The exclusion criteria were as follows: (a) severe morpho-structural alteration of the lower limbs (e.g., significant bone deformities or previous complex fractures), (b) severe neurological and vascular pathologies affecting the limb (e.g., severe peripheral neuropathy or critical limb ischemia), and (c) previous arthroplasty at the contralateral knee.

### 2.2. Surgical Technique and Prosthesis

Surgery was performed by the same experienced surgeon with an anterior midline incision and a medial parapatellar capsulotomy. The posterior cruciate ligament was sacrificed to standardize the procedure and to accommodate the design of the second-generation MP TKA; this approach improves stability through retained ligament balancing and aligns the implant on the mechanical axis [12]. For all surgeries, a mechanical alignment was adopted. All implants were the cemented second-generation MP TKA Evolution Knee System (MicroPort Orthopaedics Inc., Arlington, TN, USA) [13]. The patients underwent rehabilitation according to standard good clinical practice [14,15].

### 2.3. Clinical and Radiological Analyses

The patients were evaluated the day before surgery and after 6 months post-operatively from a clinical and functional point of view through the use of 3 different scores: the clinical and functional Knee Society Score (KSS), the Knee Injury and Osteoarthritis Outcome Score (KOOS), and the Visual Analogue Scale (VAS), the last of which was used specifically to assess pain levels. The clinical and functional KSS is a validated and sensitive method for assessing objective and subjective outcomes after a total knee arthroplasty. The KSS ranges from 0 to 100, and a score over 69 is considered a good result [16]. The KOOS is considered as a total score averaged across all domains and as individual domains (KOOS, 0 = worst condition, 100 = best condition), and the VAS scores during activity range from 0 to 10, where 0 means no pain, and 10 means the worst pain [17,18].

Radiographic scores, including the hip–knee–ankle angle (HKA), posterior tibial slope (PTS), and anatomical-mechanical angle (AMA) values, were collected before and after the total knee replacement on the full-length lower limb and lateral knee X-rays [19]. Each patient’s knee range of motion (ROM) was also assessed pre- and post-operatively by the same first operator surgeon using an analog goniometer, with the values rounded to the nearest degree [20].

### 2.4. Gait Analysis

**Figure 1:**
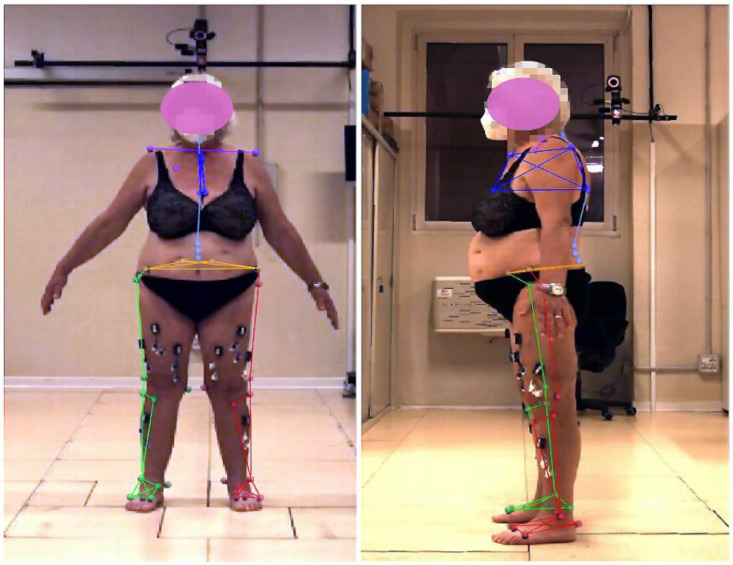
Frontal and lateral pictures of a representative patient during the collection of static pose data. The positions of the markers were used to define the anatomical reference frames [18,19,20]. Specifically for hip, knee, and ankle motion data extraction, pelvis markers were located on the left and right superior anterior–posterior iliac spines; thigh markers were located on the greater trochanter and medial and lateral epicondyles; shank markers were located on the head of the fibula, the tibial tuberosity area, and medial and lateral malleoli; and foot markers were located on the calcaneal insertion of the Achilles tendon, as well as on the first, second, and fifth metatarsal heads.

The patients were evaluated before surgery and at the six-month follow-up. Gait analysis data were collected during five repetitions of flat walking using a nine-camera motion-capture system (Vicon^®^, Nexus motion-capture Software v.2.12.1, combined with B10 Bonita Optical cameras, Oxford, UK), together with two force platforms (Kistler^TM^, model 9291B, Winterthur, CH, Switzerland) and a wireless EMG system (Zerowire, Comet, Milan, Italy) detecting the myoelectric activity of the major muscles of the lower limbs. A well-established protocol, IOR-gait (Figure 1), was used to place reflective markers on the skin at well-defined anatomical locations and to calculate the kinematics and 3D kinetics of the hip, knee, and ankle joints [21,22,23]. Calibration via six additional markers on the medial epicondyles, medial malleoli, and second metatarsal heads was performed in a supplementary single static acquisition. International recommendations were used to define the anatomical reference frames and joint rotations [24].

### 2.5. Data Processing and Statistical Analysis

Considering the proposed power of 80% and a level of 0.05 for radiographical evaluations, a sample size of at least 15 was needed to derive significant differences between the planned and post-operative HKA alignment data. This computation assumes that the general mean difference between the two conditions was 3.0 in the radiological evaluations, with the relevant standard deviation being equal to 3.0.

Pre- and post-operative 3D kinematics and kinetics of the hip, knee, and ankle joints from the gait analysis were calculated in degrees (°) and as a % of the patient’s body weight (BW) for height (H) and versus a % of the gait cycle. All data are reported in terms of the mean ± standard deviation. These values were compared with the reference data pertaining to healthy populations. The reference data were obtained from the literature and only pertained to the task of flat walking. Statistical parametric mapping (SPM) was used to assess the presence of statistically significant differences between the post-operative and normal data [22,25]. This technique, originally used to identify the regionally specific effects in neuroimaging data, allows for the analysis of continuous data in space and time and has recently been applied to biomechanical analyses [26]. Pearson’s moment–product correlation coefficient (R) was calculated to determine the relationships between the pre- and post-operative clinical and radiological scores. In all comparisons, a *p*-value of less than 0.05 was taken to reveal the statistically significant differences; otherwise, non-significance (NS) was indicated. Those performing calculations were blinded to the pre-/post-operative timeframes (Matlab^®^, R2022a The MathWorks, Inc., Natick, MA, USA).

Differences in the patient-reported outcome measures (PROMSs) and radiographic scores pre- and post-surgery were assessed through a paired Student’s *t*-test. The mean difference with 95% confidence intervals is presented alongside the *p*-values.

## 3. Results

### 3.1. Clinical Outcomes, Radiographic Score, and ROM

Significant improvement was found in all PROMSs from the pre-operative period to the 6-month follow-up stage (*p* < 0.001, Table 2).

The pre-operative VAS score, assessing pain, was found to be 6.9 ± 1.8, and the post-operative value was found to be 2.0 ± 1.9 (*p* < 0.001). The functional KSS and clinical KSS values improved, shifting from the mean pre-operative values of 51.7 ± 17.3 and 45.3 ± 16.2 to 84 ± 18.4 and 74.1 ± 12.6, respectively (*p* < 0.001 for both results). KOOS was 33.9 ± 11.8 pre-operatively and improved to 69.1 ± 16.5 at the follow-up stage (*p* < 0.001).

All radiographic scores also significantly improved (*p* < 0.038). For example, HKA increased from 174.1° ± 5.3° to 179.7° ± 0.7° on average (Table 2). Mean flexion improved from 99.4° ± 26.2° before surgery to 127.9° ± 6.8° at the follow-up.

### 3.2. Kinematic Parameters

**Figure 2:**
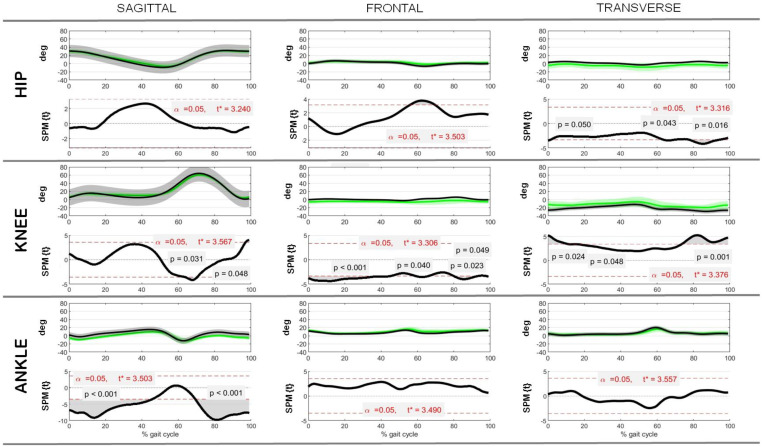
Hip, knee, and ankle joint kinematics in the sagittal, frontal, and transverse planes as a function of % gait cycle. Control and post-operative rotations (in degrees) are reported as mean ± standard deviations over all patients analyzed, along with the associated comparison of control–post-operative data via SPM (SPM{t}), where t* indicates the critical value of t given the α significance level.

**Figure 3:**
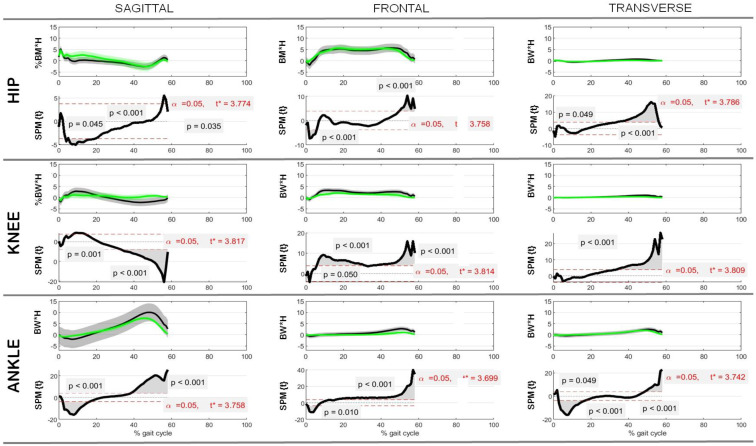
Hip, knee, and ankle joint kinetics in the sagittal, frontal, and transverse planes as a function of % gait cycle. Control and post-operative rotations (in %BW × H) are reported as mean ± standard deviations over all patients analyzed, along with the associated comparison of control–post-operative data via SPM (SPM{t}), where t* indicates the critical value of t given the α significance level.

Our comparison between the post-operative rotation data of the study cohort, the control data obtained from the literature [27], and the corresponding pre-operative data, i.e., nonphysiological data, showed few remarkable differences in terms of the overall ranges (max–min values) calculated over the entire gait cycle, whereas differences were more abundant when considering specific gait cycle timings, as in the SPM analysis (Table 3 and Figure 2 and Figure 3). Specifically, in the sagittal plane only, the post-operative knee rotation range increased significantly by an average of 6.0 ± 5.0° compared with the pre-operative data (R = 0.4, *p* = 0.04), moving in the direction of more physiological values; no other significant changes were observed between the pre- and post-operative data, including all hip and ankle joint rotations, although significative differences were observed between the post-operative and control data. On the other hand, the SPM analysis between the post-operative data and reference control data revealed significant differences in the initial and final 20% of the gait cycle for the rotation of the knee in the frontal and transverse planes and the rotation of the ankle in the sagittal plane, showing incomplete physiological restoration of lower limb kinematics after surgery.

### 3.3. Kinetic Parameters

Regarding the joint kinetics, observations similar to those for the kinematics were made. Specifically, in the transverse plane only, the post-operative ankle moment range increased significantly by an average of 0.5 ± 0.4% BW × H compared with the pre-operative data (R = 0.4, *p* = 0.03), moving in the direction of more physiological values; no other significant changes were observed between the pre- and post-operative data, including all hip and knee joint moments, although differences were observed between the post-operative and control data. On the other hand, the SPM analysis between the post-operative and reference control data revealed significant differences over the gait cycle for joint moments at the knee and the ankle in the frontal and transverse planes, as well as for the hip in the transverse plane only, showing an incomplete physiological restoration of lower limb kinetics after surgery.

## 4. Discussion

The most important finding of this present study is that normal gait kinematics were not restored 6 months after mechanically aligned second-generation MP TKA. Although, through the gait analysis, an improvement in all parameters at the follow-up compared to the pre-surgery period was noted, where the results showed that significant differences still existed in the lower limbs of the operated patients when compared to the healthy non-operated population. Particular attention was paid to the rotational data of the lower limb; a recent study by Miura et al. used gait analysis to examine the rotational movement of the knee in the last degrees of extension, defining it as the “screw home mechanism”, although to study this phenomenon, gait analysis is not the most appropriate method because dynamic radiographic studies would be required to assess it correctly [7]. The SPM analysis between the post-operative and reference control data revealed significant differences in the initial and final 20% of the gait cycle for the rotation of the knee in the frontal and transverse planes, demonstrating how the rotation of the knee is different from normal in the same degrees in which the screw home mechanism should occur. Beach et al., in a study featuring gait analysis with three different TKA designs (PS, CR, and MP), showed how the majority of patients in all their groups produced a paradoxical tibial internal rotation during terminal extension, in contrast to the physiological “screw home” kinematics [4]. This suggests that the search for a more physiological design is more complex than measuring the rotational range of motion, as all implant designs involve different or even reversed kinematics.

Moreover, in their study, Indelli et al. found that those who underwent bi-cruciate-substituting TKA experienced a significant loss of external rotation upon heel strike [28]; according to the authors, this result could be related to the absence of the anterior cruciate ligament, which contributes to the external orientation of the tibia in full extension [29].

Miura et al. enrolled 40 patients with the new-generation MP-type TKA, 20 with cruciate-substituting TKA (MP-CS group), and 20 with posterior-stabilized TKA (MP-PS group), and measured (via the use of a three-dimensional motion analysis system) the kinematics and center of axial rotation during overground walking [7]. They found that both MP-CS and MP-PS did not induce rotational motion centered on the medial ball-in-socket component during walking; rather, translational and lateral pivoting motions were observed. In contrast, Gray et al. demonstrated that the second-generation MP TKA led to the center of rotation in the transverse plane, being clearly in the medial compartment, like that reported for the healthy knee in normal walking [30]. The power of this study lies in the fact that it used gait analysis in conjunction with a mobile biplane radiographic imaging system to examine patients implanted with the MP (*n* = 26), PS (*n* = 23), and CR (*n* = 25); the authors of this study also described greater external rotation with the MP TKA design when compared to the PS design.

Our analysis of kinetic parameters showed that the three large joints of the lower limb exhibited an incomplete physiological restoration of kinetics after a knee replacement with the second-generation MP design; specifically, significant differences for the knee and ankle in the frontal and transverse planes and hip in the transverse plane only were found. This result confirms our initial hypothesis that the mechanically aligned second-generation MP TKA does not enable full restoration of physiological motion via the knee and the neighboring joints during walking.

The second-generation MP TKA is designed with features aimed at improving the replication of natural knee kinematics, as exemplified by the asymmetrical tibial base and the anterior and posterior medial lips on the tibial insert. Despite these advancements, our study shows that this design does not fully restore normal gait biomechanics, particularly in terms of knee rotational movements during level walking. This can be attributed to several factors; one key issue is that, despite improvements, the prosthesis design may still impose limitations on the knee’s ability to achieve the full range of natural movements, particularly during dynamic activities. In addition, if surgery involves the sacrifice of the posterior cruciate ligament (PCL) to improve soft tissue balance and alignment, this may have a negative impact on rotational stability. Another contributing factor is the mechanical alignment technique, which, while effective for many patients, might not perfectly align with the natural anatomy of every individual’s knee, leading to minor discrepancies in joint movement. Furthermore, patient-specific variations, such as differences in anatomy and the degree of soft tissue adaptation after surgery, can also influence the outcomes.

Although the second-generation MP design failed in trying to re-establish the native articular biomechanics of the knee, as also found by Dabirrahmani et al., there was a clear orientation towards characteristics similar to what a typical healthy knee would present [31].

This present study demonstrates that the second-generation MP TKA can achieve favorable mid-term clinical outcomes. The results are consistent with the existing literature on TKA outcomes regardless of the alignment used, as demonstrated by the patient satisfaction results [9,32,33]. Therefore, despite the differences in knee and ankle joint rotation that do not enable the second-generation MP TKA to achieve the native kinematics of the lower limb, the results show that the enrolled subjects are satisfied, and consequently, we can conclude that the implantation of this type of prosthesis with mechanical alignment produces a high clinical benefit in patients.

This study has several limitations, the first of which relates to the low number of patients recruited for the study; although a very accurate gait analysis method, combined with an advanced statistical method, was used, a larger sample number would certainly have increased the power of this study. Considering that there are several kinematic studies with relatively small cohorts, the authors believe that this work may provide useful information for future studies on kinematics after TKA.

The second limitation is related to the interposition of skin and other soft tissues between the markers and the underlying bones during the execution of 3D gait analysis. This introduces artifacts into the results, although the potential for this type of bias was reduced because the same protocol was used and the same examiner assembled the markers before and after surgery. Another limitation of this study is its short follow-up period. A follow-up at 6 months may not adequately capture the long-term outcomes and the durability of the implant.

New studies will be needed to investigate the restoration of the native kinematics of the lower limb using dynamic radiographs in addition to gait analysis, which could lead to a more accurate assessment of all the movements of the joints involved. On the other hand, the use of mechanical alignment in this study makes it necessary to evaluate the same type of second-generation prosthesis implanted with kinematic alignment and then compare the results derived from these prostheses in order to investigate how using the alignment approach in surgery can influence the restoration of native kinematics.

## 5. Conclusions

This study demonstrates that the use of new-generation MP TKA designs with mechanical alignment does not restore normal gait biomechanics. Although the values for some motor functions are similar, there are still important differences from healthy persons, such as differences in rotational knee movements in the last 20–30° of extension. The data, therefore, suggest that improvements in surgical techniques and prosthetic designs are needed to achieve normal gait kinematics after knee replacement surgery.

## Figures and Tables

**Table 1:** Physical variables. Kg: kilograms; cm: centimeters; BMI: body mass index; M: male; F: female.

Demographic Data	
Age (years)	51.7 ± 17.3
Weight (Kg)	45.3 ± 16.2
Height (cm)	6.9 ± 1.8
BMI	33.9 ± 11.8
Gender	7M/8F

**Table 2:** Patient-reported outcome scores (PROMSs) and radiographic scores collected before and after the total knee replacement. Note: CI = confidence interval.

PROMSs	Pre-Op	Post-Op	Mean Diff.	95% CI Diff.	*p*-Value
ROM	99.4° ± 26.2°	127.9° ± 6.8°	28.5°	[17.0; 26.7]	<0.001
KSS functional	51.7 ± 17.3	84 ± 18.4	32.3	[19.7; 44.9]	<0.001
KSS clinical	45.3 ± 16.2	74.1 ± 12.6	28.7	[19.3; 38.2]	<0.001
VAS	6.9 ± 1.8	2.0 ± 1.9	−4.9	[−6.4; −3.3]	<0.001
KOOS	33.9 ± 11.8	69.1 ± 16.5	35.2	[25.8; 44.6]	<0.001
Radiographic score (°)					
HKA	174.1 ± 5.3	179.7 ± 0.7	5.6	[3.6; 7.5]	0.004
AMA	7.0 ± 0.9	6.6 ± 1.0	−0.4	[−0.7; −0.1]	0.012
PTS	5.9 ± 3.3	2.8 ± 3.1	−3.1	[−6.0; −0.2]	0.038

**Table 3:** Overall excursion values (max–min) throughout the gait cycle for hip, knee, and ankle joint kinematics (in degrees) and kinetics (in %BW × H) in the sagittal, frontal, and transverse planes. The data shown are the mean ± standard deviations for all patients analyzed.

		Sagittal Plane			Frontal Plane			Transverse Plane		
		Control	Pre-Operative	Post-Operative	Control	Pre-Operative	Post-Operative	Control	Pre-Operative	Post-Operative
Hip	Rotations	41.3 ± 5.5	38.2 ± 5.1	38.7 ± 5.2	13.3 ± 3.5	8.9 ± 2.3	8.4 ± 2.1	8.4 ± 8.9	11.2 ± 4.9	12.8 ± 8.1
	Moment	7.9 ± 1.2	8.0 ± 2.6	7.7 ± 2.2	7.7 ± 1.1	7.1 ± 1.5	6.8 ± 1.5	1.3 ± 0.2	0.9 ± 0.4	0.8 ± 0.2
Knee	Rotations	63.3 ± 5.3	52.6 ± 7.1	57.6 ± 5.0	8.5 ± 3.9	10.1 ± 5.1	10.6 ± 4.2	17.1 ± 1	16.7 ± 5.8	19.7 ± 7.8
	Moment	4.2 ± 1.2	3.9 ± 1.4	3.6 ± 0.7	4.2 ± 0.9	3.6 ± 1.4	3 ± 1.0	1.1 ± 0.2	0.8 ± 0.3	0.5 ± 0.3
Ankle	Rotations	27.7 ± 4.6	25.1 ± 5.5	24.6 ± 4.0	9.3 ± 5.6	12.1 ± 3	13.1 ± 2.9	19.1 ± 5.2	15.4 ± 5.6	17.6 ± 5.4
	Moment	12.0 ± 0.9	7.9 ± 1.1	8.4 ± 0.7	3.4 ± 0.6	1.5 ± 0.5	1.4 ± 0.6	2.9 ± 0.4	1.8 ± 0.6	2.2 ± 0.3

## Data Availability

The data presented in this study are available upon reasonable request to the corresponding author. The data are not publicly available due to privacy reasons.
